# Nutritional, health and socio-demographic determinants of anaemia in adolescent girls in Kumbungu District, Ghana

**DOI:** 10.1186/s40795-023-00749-2

**Published:** 2023-07-21

**Authors:** Anthony Wemakor, Matilda Kwaako, Adinan Abdul-Rahman

**Affiliations:** Department of Nutritional Sciences, School of Allied Health Sciences, University for Development Studies, P. O. Box TL 1883, Tamale, Tamale Ghana

**Keywords:** Anemia, Adolescents, female, Nutrition determinants, Determinants of Health, Social Determinants of Health, Kumbungu, Ghana

## Abstract

**Background:**

Anaemia is a serious health problem among adolescent girls in Ghana. The aims of this study were to measure the prevalence and identify the nutritional, health, and socio-demographic determinants of anaemia in adolescent girls in Kumbungu District, Northern Region, Ghana.

**Method:**

An analytical cross-sectional study involving 370 adolescent girls residing in Kumbungu district, selected using multi-stage sampling procedure, was conducted. A semi-structured questionnaire, 24-hr dietary recall, food frequency questionnaire, Food Insecurity Experience scale, and anthropometry were used to gather information on socio-demographic characteristics, nutrition knowledge, dietary diversity score, food consumption score, food consumption frequency, household food insecurity, and waist and hip circumferences. Haemoglobin was measured using a portable HemoCue hg 301 + Analyzer. Anaemia in the adolescent girls was defined as haemoglobin concentration less than 12 g/dl. Chi-square test and binary logistic regression analysis were used to identify the determinants of anaemia.

**Results:**

The mean (± SD) age was 13.95 (± 2.94) years, and the majority of the girls were in school (79.5%) and lived in a rural area (81.1%). The mean (± SD) haemoglobin was 11.27 (± 1.19) g/dl, and 74.6% of the respondents had anaemia, with 1.6% having severe anaemia. The health determinant of anaemia was frequency of feeling nervous in the past 6 months [Adjusted Odds Ratio (AOR): 2.12: 95% Confidence Interval (CI): 1.17–3.89; p: 0.014], and the socio-demographic determinants were residential community status (AOR: 0.42; 95% CI: 0.24–0.75; p: 0.003), and fathers’ educational qualification (AOR: 2.57, 95% CI: 1.17–5.65, p: 0.019). No nutritional determinants of anaemia were identified for this study population.

**Conclusion:**

The prevalence of anaemia was very high and the frequency of feeling nervous in the past 6 months, residential community status, and fathers’ educational qualification were associated with anaemia among adolescent girls in Kumbungu district, Ghana. The prevalence of anaemia measured highlights the need for intensification of anaemia prevention and management interventions in the district.

**Supplementary Information:**

The online version contains supplementary material available at 10.1186/s40795-023-00749-2.

## Introduction

Anaemia in adolescence is a problem of public health significance. Globally, anaemia prevalence was estimated at 29.4% among females in their reproductive age by World Health Organization (WHO) [1]. About 50% of adolescent girls living in Sub-Saharan Africa are anaemic [2], and anaemia prevalence of 26.4% was estimated among non-pregnant adolescent girls aged 15 to 19 years in Ghana [3]. Anaemia reduces the ability of blood to transport oxygen throughout the body [4] and results in reduced immunity, impaired physical performance and poor neurodevelopment in adolescence [5, 6].

The main cause of anaemia is inadequate dietary iron intake resulting from consuming iron-poor foods. Among adolescents, anaemia is most frequently caused by nutritional deficiencies due to rapid growth and physical changes, high iron requirements associated with adolescence, and high infection and worm infestation rates [7, 8]. According to a number of research studies, the individual determinants of anaemia in adolescent girls include nutritional status, dietary diversity score, age, educational status, marital status, occupation, religion, wealth status, and socio-economic status, while the household-level determinants include food insecurity, toilet facility type, drinking water source, and proximity to health facility [9–12]. Apart from the individual and household characteristics, area level characteristics such as residential area classification (rural, peri-urban, urban) of adolescent girls also influence their risk of anaemia [11, 13].

To address the high number of cases of anaemia among adolescent girls in the country, Ghana commenced the first phase of weekly Girls’ Iron-Folic Acid Tablet Supplementation (GIFTS) programme for girls aged 15–19 years in Volta, Bono, Ahafo, Upper East and Northern regions in December 2018 [14]. The GIFTS Programme provides free weekly Iron-Folic Acid (IFA) supplements to adolescent females in school and out of school in an effort to raise their haemoglobin levels and reduce anaemia risk. A longitudinal study conducted on the participants of GIFTS revealed that supplementation with IFA increased haemoglobin levels and reduced the risk of anaemia [15].

In most developing countries including Ghana, anaemia prevention interventions are mostly targeted to infants, young children, pregnant women, and lactating women leaving adolescents to their fate. As with the other indicators of malnutrition, the Northern Region bears the most burden of anaemia, 64.6% of adolescent girls were estimated to be having anaemia [16]. However, little is documented on prevalence and determinants of anaemia among adolescent girls in the Kumbungu District in the Northern Region. This study sought to fill this knowledge gap and contribute to evidence base on anaemia in the district. The objectives of this study were to assess the prevalence and identify nutritional, health and socio-demographic determinants of anaemia in adolescent girls in Kumbungu District, Ghana.

## Methods

### Study design, site, population and subjects

An analytical cross-sectional study involving adolescent girls in Kumbungu district, Northern region, Ghana was carried out. The Kumbungu District is in the Northern region and has an estimated population of 46,171 and a population density of 89 people per square kilometer. Of this population, 13,631 are adolescents comprising 75% males and 25% females who attend school [17]. Dagombas are the indigenous people making up about 95% of the district’s population; however, persons of Gonja and Ewe ethnicities engage in fishing activities along the White Volta. The predominant religions practised are Islamic and traditional religions, but there are pockets of Christians throughout the district. The study was conducted among adolescent girls in six communities in two selected sub-districts. The six communities are Wuba, Gbugli and Zangbalun in Dalun sub-district and Gumo, Kanfehiyili and Cheshegu in Gupanarigu sub-district.

### Sample size and sampling technique

Sample size was determined using single population proportion formula [18]. Using a critical value of 95% confidence level of 1.96, prevalence of anaemia in adolescent girls in Northern Region 64.6% [16], and margin of error of 0.05, a minimum sample of 370 was estimated. Probability proportional to size was used to determine the sample size for each of the 6 communities that participated in the study i.e., 60 participants were selected from each of the five communities and 70 participants in the remaining one community. Both the communities and subjects were selected using simple random sampling. On each data collection day, balloting was done with ‘yes’ or ‘no’ written on pieces of papers, folded into a container, and the participants were allowed to pick. Any participant who selected ‘yes’ and consented to participate in the study was interviewed.

### Data collection

Data were collected in March and April, 2022. A semi-structured questionnaire, 24-hr dietary recall, food frequency questionnaire, Food Insecurity Experience scale, and anthropometry were used to gather information on socio-demographic characteristics, nutrition knowledge, dietary diversity score, food consumption score, food consumption frequency, household food insecurity, and waist and hip circumferences. The nutrition knowledge and IFA practices sub-scale of the investigator-constructed semi-structured questionnaire underwent content and face validations. Content validation was carried out when composing the statements used to measure the girls’ nutrition knowledge and IFA practices in a focus group discussion by a team of ANC nurses who were conversant with the education given to women on nutrition, iron, folic acid, and anaemia in pregnancy in antenatal clinics in Ghana. Face validation involved pretesting the questionnaire on adolescent girls in another community and revising unclear questions until the girls were satisfied that the questions could adequately measure their nutrition knowledge and IFA practices. The Cronbach’s alpha for the nutrition knowledge sub-scale is 0.76. The questionnaires were presented in face-to-face interviews with adolescent girls in their homes in English language or the local language spoken in the study area (Dagbani). Haemoglobin was measured using a portable HemoCue hg 301 + Analyzer [19]. 10 µL capillary blood sample was taken by pricking the tip of the index finger with a sterilized disposable lancet and the blood was put on the optical window of the micro cuvette through capillary action. The displayed haemoglobin level was observed and recorded. Participants’ waist and hip circumferences were measured to the nearest 0.1 cm using a tape measure. Waist circumference was measured at the mid-point (navel), and hip circumference was measured at the maximum circumference of the hip in a horizontal plane. The interview took 20 min on the average to complete per study participant. At the end of the day during the fieldwork, the completed questionnaires were checked by supervisors to ensure that all questions were answered. The data were collected by 4 research assistants (including a candidate for MPhil Public Health Nutrition and a phlebotomist) and two lecturers of the Department of Nutritional Sciences, School of Allied Health Sciences, University for Development Studies, Tamale, Ghana. Prior to the data collection exercise, there was a 3-day training workshop to enable the enumerators understand the questions and to sharpen their data collection skills.

### Study questionnaires and definition of variables

#### Anaemia

Anaemia in adolescence was the outcome variable and was defined as haemoglobin concentration less than 12 g/dl. The haemoglobin level was further categorized based on WHO classification as normal (≥ 12 g/dl), mild anaemia (11.0-11.9 g/dl), moderate anaemia (8.0-10.9 g/dl) and severe anaemia (< 8 g/dl) [20].

#### Household food security

Food insecurity was assessed using Food Insecurity Experience Scale (FIES). The FIES was established by Food and Agriculture Organization Voices of the Hunger for estimating food insecurity prevalence. FIES is a food insecurity severity experience matrix that depends on immediate responses of respondents to questions about their access to sufficient food. The eight questions in this scale required respondents to answer ‘yes’ (scored 1) or ‘no’ (scored 0) concerning their access to sufficient food for the past one year. The scores were aggregated (plausible range 0–8), and the raw score was used to classify the households into food secure (raw score ≤ 3) and food insecure (raw score ≥ 4) categories [21].

#### Minimum dietary diversity-women

The respondents were asked to recall the foods and drinks they had consumed in the previous 24-hr before the interview. Based on the information provided, it was determined if they had eaten from the 10 food groups or not irrespective of the quantities [22]. The food groups are grains, roots and tubers; meat, poultry and fish; dairy; eggs; pulses; nuts and seeds; dark green leafy vegetables; other vegetables; other fruits; and other vitamin A-rich fruits and vegetables. For each food group they ate from they got a score of “1” otherwise a score of “0”. The individual dietary diversity score was calculated by summing up all the ten food groups to get the overall score for each participant (plausible range 0–10). Respondents who consumed at least five of the ten food groups met the minimum dietary diversity-women criterion and those who consumed less than five food groups did not [22]. Minimum dietary diversity-women is a measure of access to micronutrient-rich foods.

*Food consumption score*: The number of days foods were consumed from eight food groups in a 7-day period by the respondents was recorded, each food group frequency was multiplied by the food group weight, and the scores added up [23]. The overall score ranges potentially ranges from 0 to 112. The food groups are main staples; pulses; vegetables; fruits; meat, egg, and sea food; milk; sugar; and fats and oils and the food group weights are 2, 3, 1, 1, 4, 4, 0.5, and 0.5 respectively. The composite score was divided into three categories of food consumption: poor (0.0–21.0), borderline (21.5–35.0), and acceptable (> 35.0) [23].

#### Waist-to-hip ratio

The waist-to-hip ratio was calculated by dividing waist circumference by hip circumference, and the respondents whose waist-to-hip ratio was more than 0.85 were classified as having abdominal obesity, otherwise they were classified as not having abdominal obesity [24, 25].

#### Physical activity

The question *“Over the past 7 days, on how many days were you physically active for a total of at least 60 minutes per day?”* [26] was used to measure physical activity level. Respondents who were physically active for at least 5 days were classified as physically active, otherwise they were classified as physically inactive.

#### Nutrition knowledge index

Nine statements on general nutrition and anaemia were read out to the respondents and they were expected to determine if each statement was true or false, Supplementary File S1. These statements were on importance of dietary diversity for good health; benefits of iron-rich foods, and fruits; signs and symptoms of iron deficiency; and consequences of iron deficiency anaemia. The scores for the nine statements were aggregated and the sum ranging from 0 to 9 used to classify them into two categories using the natural mean of the score (4.5).

#### Practices on iron-folic acid

Seven questions bothering on participation in the GIFTS programme, whether the IFA supplement was taken when missed, the experiences of the respondents after taking the IFA supplement, whether their families encouraged them to take the IFA supplement, and their perceptions on the IFA supplements were explored.

#### Malaria and worm infestation

The participants were asked if they had experienced malaria and/or worm infestation in the last 6 months prior to the survey and if they did whether they received treatment.

*Self-rated health*: The question: *“In general would you say that your health is excellent, very good, good, moderate, bad or very bad?”* was used to measure self-rated health [27]. The responses were grouped into two categories (1) Good/very good/excellent and (2) Very bad/bad/moderate.

*General health*: General health was measured using the question: “*In the past 6 months, have you had the following problems: headache, stomach ache, backache, feeling low, feeling irritable or bad tempered, feeling nervous, difficulties in getting to sleep and dizziness?”* The five responses were grouped into (1) Rarely or seldom, (2) Sometimes and (3) Fairly/very often.

#### Socio-demographic and economic characteristics of respondents

Residential community status was classified as rural or peri-urban, age was measured as a continuous variable but grouped as 10–14 years and 15–19 years, and educational level was categorized into “no education”, “primary school”, “junior high school”, and “secondary/vocational school”. The religion practised, marital status and ethnicity were categorized into two levels each i.e., “Christian” and “Islam”, “single, never married” and “married”, and “Dagomba” and “others” respectively. The occupation of parents was categorized into “farmer”, “trader” and “others”. The educational status of the parents were “no education”, “primary school”, “junior high school”, “secondary/vocational school”, and “Higher National Diploma and above”. Household size was grouped into 3–6, 7–10, and 11+.

#### Perceived socio-economic status

The socio-economic status of the families was measured using a question from Healthy Behaviours in School aged Children survey *“How well off do you think your family is?”* [28]. The five response options were grouped into three as low (“not at all” and “not particularly”), middle (“fairly”) and high (“rather” and “very”).

### Statistical analysis

Stata 15 IC (Stata Corp) was used to analyze the data. Descriptive statistics (frequencies and percentages for categorical variables and means and standard deviations for continuous variables) were used to present the results. Chi-square test and logistic regression modelling were used to identify the nutritional, health and socio-demographic determinants of anaemia. The factors significantly associated with anaemia in bivariable analyses were then entered simultaneously into a multivariable logistic regression model to identify the independent determinants of anaemia in adolescent girls. P-value < 0.05 was regarded as statistically significant. Model fit was evaluated using Hosmer-Lemeshow goodness of fit test.

## Results

### Socio-demographic and economic characteristics of respondents

The mean (± SD) age of the respondents was 13.95 (± 2.94) years and majority were between the ages of 10 and 14 years (55.7%). The majority of the respondents lived in rural areas (81.1%), were students (79.5%), had no mobile phone (84.6%), and belonged to the low perceived socio-economic status index (64.6%), Table 1. The vast majority of respondents, 97.8% were single or never married, 98.9% belonged to the Dagomba tribe, and 85.7% practised Islam. The majority of the fathers (84.9%) and mothers (52.4%) of the respondents were farmers, and also majority did not have any form of formal education (father 80.0%, mother 87.0%).

**Table 1:** Socio-demographic characteristics of respondents

Characteristic	Frequency	Percent
***Status of community***		
Rural	300	81.1
Peri-urban	70	18.9
***Age group (years)***		
10–14	206	55.7
15–19	164	44.3
***Education***		
No education	77	20.8
Primary school	154	41.6
Junior High School	109	29.5
Secondary/vocational school	30	8.1
***Religion***		
Christian	53	14.3
Islam	317	85.7
***Marital status***		
Single, never married	362	97.8
Married	8	2.2
***Ethnicity***		
Dagomba	368	98.9
Others	2	1.0
***Occupation of mother***		
Farmer	194	52.4
Trader	153	41.4
Others	23	6.2
***Education of father***		
No education	296	80.0
Primary school	27	7.3
Junior High School	17	4.6
Secondary/vocational school	22	5.9
Higher National Diploma and above	8	2.2
***Education of mother***		
No education	322	87.0
Primary school	21	5.7
Junior High School	17	4.6
Secondary/vocational school	9	2.4
Higher National Diploma and above	1	0.3
***Occupation of father***		
Farmer	314	84.9
Trader	27	7.3
Others	29	7.8
***Household size***		
3–6	92	24.9
7–10	184	49.7
11+	94	25.4
***Perceived socio-economic status***		
Low	239	64.6
Middle	110	29.7
High	21	5.7
***Respondent has a phone***		
No	313	84.6
Yes	57	15.4

### Consumption from food groups and food frequency

Almost all the respondents (99.5%) ate grains, roots, and tubers, while only 8.6% each ate dairy and other fruits (Table 2). The vast majority of respondents (95.9%) consumed flesh foods (meat, poultry, and fish) and other vegetables (97.6%), while less than one-fifth consumed pulses (beans, peas, and lentils) (13.8%), and eggs (4.1%). Dark green leafy vegetables (58.6%), and other vitamin A-rich fruits and vegetables (44.1%) were consumed by about half of the subjects.

**Table 2:** Percentage of respondents who ate from the 10 food groups in the previous 24 h before survey

Food group	Frequency	Percent
Grains, roots and tubers	368	99.5
Other vitamin A-rich fruits and vegetables	163	44.1
Meat, poultry and fish	355	95.9
Dairy	32	8.6
Dark green leafy vegetables	217	58.6
Other vegetables	361	97.6
Other fruits	32	8.6
Eggs	15	4.1
Pulses	51	13.8
Nuts and seeds	169	45.7

According to a 7-day food frequency questionnaire, 75.1% of respondents consumed staples on more than 5 days per week, while 61.1% consumed pulses on 1–3 days per week (Table 3). Vegetables were consumed by 51.1% of respondents for 4–5 days per week. Meat, eggs, and seafood were consumed by 48.6% of respondents on 4–5 days per week. Only 25.9% of respondents consumed milk on 1–3 days per week, while sugar was consumed on > 5 days per week by 64.6% of respondents. The majority (48.1%) of respondents consumed fats and oils for 1–3 days per week, while about one-third (33.8%) consumed beverages 1–3 times per week.

**Table 3:** Food frequency in the last 7 days

Variable	Frequency	Percent
***Main staples***		
Never	1	0.3
1–3 days	5	1.4
4–5 days	86	23.2
> 5 days	278	75.1
***Pulses***		
Never	82	22.2
1–3 days	226	61.1
4–5 days	50	13.5
> 5 days	12	3.2
***Vegetables***		
Never	1	0.3
1–3 days	25	6.8
4–5 day	189	51.1
> 5 days	155	41.9
***Fruits***		
Never	78	21.1
1–3 days	220	59.5
4–5 days	47	12.7
> 5 days	25	6.8
***Meat, egg, and sea food***		
Never	6	1.6
1–3 days	71	19.2
4–5 days	180	48.6
> 5 days	113	30.5
***Milk***		
Never	257	69.5
1–3 days	96	25.9
4–5 days	10	2.7
> 5 days	7	1.9
***Sugar***		
Never	1	0.3
1–3 days	48	13.0
4–5 days	82	22.2
> 5 days	239	64.6
***Fats & Oils***		
Never	8	2.2
1–3 days	178	48.1
4–5 days	133	35.9
> 5 days	51	13.8
***Beverages (tea, coffee)***		
Never	80	21.6
1–3 days	125	33.8
4–5 days	74	20.0
> 5 days	91	24.6

### Nutritional factors and nutrition knowledge index

A greater majority of the respondents were food insecure (89.7%) but had acceptable food consumption score (81.9%) and a little more than half (57.3%) met the dietary diversity requirement by eating foods from at least five of the ten food groups, Table 4. Also, a little more than half (56.5%) of the respondents had high nutrition knowledge.

**Table 4:** Percentages of respondents for selected nutritional factors

Variable	Frequency	Percent	95% Confidence Interval
Minimum Dietary Diversity-Women (Yes)	212	57.3	52.1–62.4
Food consumption score (Acceptable)	303	81.9	77.6–85.7
Household food insecurity (Yes)	332	89.7	86.2–92.6
Nutrition knowledge index (High)	209	56.5	51.3–61.6
Participation in Girls’ Iron Folic Acid Tablet Supplementation programme (Yes)	175	47.3	42.1–52.3

### Nutritional, health and socio-demographic determinants of anaemia

The mean (± SD) haemoglobin was 11.27 (± 1.19) g/dl, and 74.6% of the respondents had anaemia, with 40.8%, 32.2% and 1.6% having mild, moderate and severe anaemia respectively. The nutritional, health and socio-demographic factors of adolescent girls were compared to their anaemia status. None of the nutritional factors of the respondents was associated with their anaemia status (Table 5). The frequency of feeling nervous in the last 6 months (p = 0.029), residential community status (p = 0.005), and fathers’ educational level (p = 0.039) were significant in both bivariable (Tables 6 and 7) and multivariable (Table 8) analyses. Respondents who felt nervousness fairly/very frequently in the previous 6 months were two times more likely to be anaemic compared to those who felt nervous rarely or seldomly [Adjusted Odds Ratio (AOR) = 2.12, 95% 95% Confidence Interval (CI): 1.17–3.89, p = 0.014]. Adolescents from peri-urban communities were 58% less likely to be anaemic compared to those from rural communities (AOR = 0.42, 95% CI: 0.24–0.75, p = 0.003). Again, adolescents whose fathers had no formal education were about three times more likely to be anaemic compared to those whose fathers had higher education (secondary/vocational school or above) (AOR = 2.57, 95% CI: 1.17–5.65, p = 0.019). With respect to the evaluation of the fit of the logistic regression model, the insignificant p-value (p = 0.92) obtained suggests that the model fitted the data well.

**Table 5:** Nutritional factors of respondents as determinants of anaemia

Variable	Total	Anaemia, No, Freq (%)	Anaemia, Yes, Freq (%)	Test statistics
***Consumption of iron-rich foods (meat, poultry and fish)***				X2 = 2.9; p = 0.089
No	15	1 (6.7)	14 (93.3)	
Yes	355	93 (26.2)	262 (73.8)	
***Frequency of consumption of iron-rich foods (meat, poultry and fish)***				X2 = 0.2; p = 0.672
≤3	77	21 (27.3)	56 (72.7)	
3+	293	73 (24.9)	220 (75.1)	
***Minimum Dietary Diversity-Women***				X2 = 0.3; p = 0.605
No	158	38 (24.1)	120 (75.9)	
Yes	212	56 (26.4)	156 (73.6)	
***Food consumption score***				X2 = 0.1; p = 0.762
Borderline	67	18 (26.9)	49 (73.1)	
Acceptable	303	76 (25.1)	227 (74.9)	
***Household food insecurity***				X2 = 2.9; p = 0.087
No	38	14 (36.8)	24 (63.2)	
Yes	332	80 (24.1)	252 (75.9)	
***Nutrition knowledge index***				X2 = 2.0; p = 0.155
Low	161	35 (21.7)	126 (78.3)	
High	209	59 (28.2)	150 (71.8)	
***Participates in Girls’ Iron-Folic Acid Tablet Supplementation Programme***				X2 = 2.4; p = 0.118
Yes	175	51 (29.1)	124 (70.9)	
No	195	43 (22.1)	152 (77.9)	

**Table 6:** Health-related variables of respondents as determinants of anaemia

Variable	Total	Anaemia, No, Freq (%)	Anaemia, Yes, Freq (%)	Test statistics
***Had malaria within the last 6 months***				X2 = 0.6; p = 0.448
Yes	217	52 (24.0)	165 (76.0)	
No	153	42 (27.5)	111 (72.5)	
***Had worm infestation within the last 6 months***				X2 = 0.0; p = 0.939
Yes	168	43 (25.6)	125 (74.4)	
No	202	51 (25.2)	151 (74.8)	
***Dewormed within the last 6 months***				X2 = 0.0; p = 0.958
Yes	133	34 (25.6)	99 (74.4)	
No	237	60 (25.3)	177 (74.7)	
***Self-rated health status***				X2 = 0.2; p = 0.624
Excellent/very good/good	197	48 (24.4)	149 (75.6)	
Moderate/poor/very poor	173	46 (26.6)	127 (73.4)	
***Started menstruation***				X2 = 0.4; p = 0.512
Yes	186	50 (26.9)	136 (73.1)	
No	184	44 (23.9)	140 (76.1)	
***Frequency of experiencing headache in the last 6 months***				X2 = 0.4; p = 0.821
Rarely or seldom	199	52 (26.1)	147 (73.9)	
Sometimes	104	27 (26.0)	77 (74.0)	
Fairly/very often	67	15 (22.4)	52 (77.6)	
***Frequency of experiencing stomach ache in the last 6 months***				X2 = 0.2; p = 0.904
Rarely or seldom	254	63 (24.8)	191 (75.2)	
Sometimes	84	22 (26.2)	62 (73.8)	
Fairly/very often	32	9 (28.1)	23 (71.9)	
***Frequency of experiencing backache in the last 6 months***				X2 = 2.4; p = 0.308
Rarely or seldom	315	83 (26.3)	232 (73.7)	
Sometimes	42	10 (23.8)	32 (76.2)	
Fairly/very often	13	1 (7.7)	12 (92.3)	
***Frequency of experiencing low feelings in the last 6 months***				X2 = 1.1; p = 0.583
Rarely or seldom	197	48 (24.4)	149 (75.6)	
Sometimes	107	31 (29.0)	76 (71.0)	
Fairly/very often	66	15 (22.7)	51 (77.3)	
***Frequency of experiencing irritability in the last 6 months***				X2 = 0.8; p = 0.657
Rarely or seldom	182	46 (25.3)	136 (74.7)	
Sometimes	65	14 (21.5)	51 (78.5)	
Fairly/very often	123	34 (27.6)	89 (72.4)	
***Frequency of experiencing nervousness in the last 6 months***				X2 = 7.1; p = **0.029**
Rarely or seldom	198	56 (28.3)	142 (71.7)	
Sometimes	58	19 (32.8)	39 (67.2)	
Fairly/very often	114	19 (16.7)	95 (83.3)	
***Frequency of experiencing difficulty in sleeping in the last 6 months***				X2 = 1.5; p = 0.469
Rarely or seldom	308	82 (26.6)	226 (73.4)	
Sometimes	39	8 (20.5)	31 (79.5)	
Fairly/very often	23	4 (17.4)	19 (82.6)	
***Frequency of experiencing dizziness in the last 6 months***				X2 = 2.8; p = 0.243
Rarely or seldom	241	67 (27.8)	174 (72.2)	
Sometimes	76	18 (23.7)	58 (76.3)	
Fairly/very often	53	9 (17.0)	44 (83.0)	
***Physically active in the last week***				X2 = 0.2; p = 0.655
No	233	61 (26.2)	172 (73.8)	
Yes	137	33 (24.1)	104 (75.9)	
***Diagnosed with anaemia in the last 3 months***				X2 = 2.1; p = 0.149
Yes	27	10 (37.0)	17 (63.0)	
No	343	84 (24.5)	259 (75.5)	
***Abdominal obesity***				X2 = 0.5; p = 0.480
No	197	53 (26.9)	144 (73.1)	
Yes	173	41 (23.7)	132 (76.3)	

**Table 7:** Socio-demographic factors as determinants of anaemia

Variable	Total	Anaemia, No, Freq (%)	Anaemia, Yes, Freq (%)	Test statistics
***Status of residential community***				X2 = 7.9; p = **0.005**
Rural	300	67 (22.3)	233 (77.7)	
Peri-urban	70	27 (38.6)	43 (61.4)	
***Education***				X2 = 1.9; p = 0.587
No education	77	18 (23.4)	59 (76.6)	
Primary school	154	40 (26.0)	114 (74.0)	
Junior High School	109	31 (28.4)	78 (71.6)	
Secondary/vocational school or above	30	5 (16.7)	25 (83.3)	
***Religion***				X2 = 0.2; p = 0.618
Christianity	53	12 (22.6)	41 (77.4)	
Islam	317	82 (25.9)	235 (74.1)	
***Ethnicity***				X2 = 0.0; p = 0.985
Dagomba	366	93 (25.4)	273 (74.6)	
Others	4	1 (25.0)	3 (75.0)	
***Fathers’ occupation***				X2 = 4.4; p = 0.111
Farmer	314	74 (23.6)	240 (76.4)	
Trader	27	11 (40.7)	16 (59.3)	
Others	29	9 (31.0)	20 (69.0)	
***Mothers’ occupation***				X2 = 2.3; p = 0.318
Farmer	194	43 (22.2)	151 (77.8)	
Trader	153	44 (28.8)	109 (71.2)	
Others	23	7 (30.4)	16 (69.6)	
***Fathers’ education***				X2 = 8.4; p = **0.039**
No education	296	66 (22.3)	230 (77.7)	
Primary school	27	9 (33.3)	18 (66.7)	
Junior High School	17	6 (35.3)	11 (64.7)	
Secondary/vocational School or above	30	13 (43.3)	17 (56.7)	
***Mothers’ education***				X2 = 0.7; p = 0.873
No education	322	83 (25.8)	239 (74.2)	
Primary school	21	5 (23.8)	16 (76.2)	
Junior High School	17	3 (17.6)	14 (82.4)	
Secondary/vocational school or above	10	3 (30.0)	7 (70.0)	
***Household size***				X2 = 3.3; p = 0.343
3–6	92	19 (20.7)	73 (79.3)	
7–10	184	52 (28.3)	132 (71.7)	
11–14	47	9 (19.1)	38 (80.9)	
15+	47	14 (29.8)	33 (70.2)	
***Perceived socio-economic status***				X2 = 0.5; p = 0.785
Low	239	62 (25.9)	177 (74.1)	
Middle	110	28 (25.5)	82 (74.5)	
High	21	4 (19.0)	17 (81.0)	
***Respondent has a mobile phone***				X2 = 0.0; p = 0.864
No	313	79 (25.2)	234 (74.8)	
Yes	57	15 (26.3)	42 (73.7)	

**Table 8:** Multivariable determinants of anaemia in adolescent girls

Characteristics	Adjusted Odds Ratio	95% Confidence Interval of AOR	P-Value
***Residential community status***			
Rural	1.00		
Peri-urban	0.42	0.24–0.75	**0.003**
***Frequency of experiencing nervousness in the past 6 months***			
Rarely or seldom	1.00		
Sometimes	0.79	0.42–1.52	0.486
Fairly/very often	2.12	1.16–3.88	**0.014**
***Fathers’ educational status***			
Beyond secondary/vocational school	1.00		
No education	2.57	1.17–5.65	**0.019**
Primary school	1.50	0.50–4.50	0.470
Secondary/vocational school	1.38	0.39–4.86	0.613

## Discussion

The determinants of anaemia in adolescent girls were studied in Kumbungu district, Ghana. A significant proportion of the subjects were identified to have anaemia (74.6%). Following bivariable and multivariable studies of putative determinants of anaemia, frequency of feeling nervous in the preceding six months, residential community status, and fathers’ educational status were found to be independent determinants of anaemia in the adolescent girls.

Anaemia is very common among adolescent girls. Globally, WHO estimated anaemia prevalence to be 29.4% among females in their reproductive age [1], and on the African continent, anaemia prevalence ranges from 11.1% [29] to 39.0% in Ethiopia [30], is 26.5% in Kenya [9], and 29% in Rwanda [31] in adolescents. The highest prevalence recorded so far on the continent (77%) from our review is in Sudan [32]. In Ghana, anaemia prevalence among adolescent females ranges from 24% [14] to 64.6% [16], and in between these are 26.4% in the Micronutrient Survey [3], 49.5% [33], and 50.3% [34]. The highest rate of 64.6% [16] was reported in adolescent girls in Northern Ghana.

The varying rates of anaemia in adolescent girls in Ghana and Africa could be attributed to differences in the amounts of iron-rich foods consumed, uptake of IFA supplementation, access to health services, and socio-demographic and economic characteristics of subjects. The high prevalence of anaemia reported in the study area could be due to persistent inadequate intake of iron-rich foods and low rate of participation in the GIFTS programme. The WHO advises all women of reproductive age (15–49 years) to take intermittent IFA supplements when the prevalence of anaemia surpasses 20% but only 47.3% of the girls in Kumbungu District participate in the GIFTS programme. A previous study has reported improved haemoglobin and low level of anaemia among participants of GIFTS [15] so the haemoglobin status of the girls would have been better if participation in the programme was better.

The general health status of the adolescent girls may have implications for the risk of anaemia. The frequency of feeling nervous in the past 6 months has been identified as a health determinant of anaemia in the study population. Teenagers who experienced nervousness frequently compared to those who did not in the preceding six months have a higher risk of anaemia. Iron is an essential element in brain metabolism and its deficiency can cause changes in neurotransmitter homeostasis, decrease myelin production, impair synaptogenesis, and decline the function of the basal ganglia leading to impaired cognitive functions and psychomotor development [5]. Iron deficiency in children is linked to poor health and serious neurological damage, including mental, motor, social, emotional, neurophysiological, and neurocognitive dysfunction [35]. In adulthood, anaemia has been associated with psychiatric disorders (depression, anxiety disorders, sleep disorders, and psychotic disorders). An epidemiological study reported an increased risk for psychiatric disorders comparing iron deficiency and non-iron deficiency groups (adjusted hazard ratio 1.52, 95% CI = 1.45–1.59) [36]. On the other hand, nervousness can have a negative impact on iron levels resulting in anaemia, as nervous individuals tend to select and consume less nutritious meals which contain less iron and vitamin C which aids in the absorption of iron. The emerging link between iron deficiency and mental illness needs further investigation and elucidation of the mechanism involved to provide a basis for prevention and treatment of anaemia.

The socio-demographic determinants of anaemia in the study population are community of residence, and the father’s educational level. The risk of anaemia is higher among the girls staying in rural areas compared to those in peri-urban areas. Similar to our finding, anaemia has been reported to be more common among adolescent girls living in rural areas than those living in urban areas in Ethiopia [11] and India [13]. This observation could be due to better economic conditions and availability of income earning opportunities for families in the urban areas compared to the rural areas, and the lack of information on nutrition by the girls staying in the rural areas. The positive association between higher levels of incomes and household food security may translate into availability of iron-rich foods in the homes and reduce the risk of anaemia in the girls in the peri-urban areas. Teenagers whose fathers had no formal education were shown to have a higher risk of anaemia compared to those whose fathers had some form of education, and a similar finding was also reported for a group of adolescent girls in Kenya [9]. However, it was also previously found that, a teenager’s risk of anaemia negatively correlates with the mother’s level of education [37, 38]. Due to the impact of education on work prospects and potential dietary effects, a parent’s educational level is likely to predict their child’s nutritional status. Therefore, the fathers’ education might have influenced how much iron was consumed, either through well-informed decisions or increased salaries making iron-rich foods (i.e., meat and fish) more readily available in the households.

The study did not identify any nutritional determinants of anaemia. While consumption of iron-rich foods, individual dietary diversity, food consumption score, household food security, participation in GIFTS programme and nutrition knowledge could be linked to anaemia status of adolescents, that is not the case for this study population. Further research is warranted to help unravel the reason for this observation. Also, since this study did not assess all nutritional determinants of anaemia, it is possible that the unmeasured nutritional determinants may be relevant in anaemia etiology in this study population.

### Strengths and limitations

This study has some strengths and limitations. By way of strength, we included a host of variables reported in previous studies to be associated to or could be reasonably linked to anaemia among adolescent girls, and haemoglobin measurement was carried out by the investigators. On limitations, other haematological parameters of iron status i.e., ferritin and total iron binding capacity were not measured; intake of iron-rich foods was not quantitatively measured; and the cross-sectional study design used could not establish a causal link between anaemia and its determinants in this study population.

## Conclusion

The prevalence of anaemia was high and the frequency of feeling nervous in the past 6 months was identified as a health determinant of anaemia while the residential community status, and fathers’ educational level were identified as socio-demographic determinants of anaemia among the adolescent girls in Kumbungu District, Ghana. None of the nutritional factors of the girls was associated to their anaemia status. The high prevalence of anaemia measured highlights the need for intensification of anaemia prevention and management interventions in the district.

## Electronic supplementary material

Below is the link to the electronic supplementary material.

Supplementary Material 1

## Data Availability

The datasets used and/or analyzed during the current study are available from the corresponding author on reasonable request.

## Abbreviations

AOR: Adjusted Odds Ratio
CI: Confidence Interval
GIFTS: Girls Iron Folic-Acid Tablet Supplementation
IFA: Iron-Folic Acid
WHO: World Health Organization
