# Supplementary material for: Nutritional, health and socio-demographic determinants of anaemia in adolescent girls in Kumbungu District, Ghana
Source: BMC Nutr. 2023 Jul 21;9:90. doi: 10.1186/s40795-023-00749-2 (PMC10362769; doi:10.1186/s40795-023-00749-2)
Supplement: Supplementary file 1 — Supplementary Material 1 [file 40795_2023_749_MOESM1_ESM.docx]

**NUTRITIONAL, HEALTH AND SOCIO-DEMOGRAPHIC DETERMINANTS OF ANAEMIA IN ADOLESCENT GIRLS IN KUMBUNGU DISTRICT, GHANA**

**SEMI-STRUCTURED QUESTIONNAIRE (SOCIO-DEMOGRAPHIC CHARACTERISTICS, NUTRITION KNOWLEDGE INDEX AND PRACTICES ON IRON-FOLIC ACID)**

**SECTION A: IDENTIFICATION**

Name of community: ………………………

Status of community: 1. Rural 2. Peri-urban 3. Urban

Serial number: ……………

**SECTION B: SOCIO-DEMOGRAPIC CHARACTERISTICS**

Instruction: Please answer the following questions by providing a response or ticking the appropriate options.

1. Age (years) ………………
2. Education
3. No Education
4. Primary
5. J.H.S
6. S.H.S/ Vocational School
7. Others (specify)…………………………….
8. Religion
9. Christianity
10. Islam
11. Traditional

4. Others (specify)……………………………..

1. Marital Status
2. Single
3. Married
4. Divorced/ Separated
5. Ethnicity
6. Dagomba
7. Gonja
8. Mamprusi
9. Others (specify) ………………………..
10. Occupation
11. Student
12. Trader
13. Farmer
14. Fishing
15. Hair dresser
16. Seamstress
17. Apprentice
18. Others (specify)………………
19. Father’s Occupation
20. Farmer
21. Fishing
22. Trader
23. Health Practitioner
24. Teacher
25. Others (specify)………………………………
26. Mother’s Occupation
27. Farmer
28. Trader
29. Health Practitioner
30. Teacher
31. Others (specify)………………………………
32. Father’s Educational Qualification
33. No Education
34. Primary School
35. JHS
36. O’ Level/ A’ Level/SHS/ Vocational School
37. HND/BSc./B.ED
38. PGD/Masters
39. Mother’s Educational Qualification
40. No Education
41. Primary School
42. JHS
43. O’ Level/ A’ Level /SHS/ Vocational School
44. HND/BSc./B.ED
45. PGD/Masters
46. How many are you in the family? ………………

**SECTION C: NUTRITIONAL KNOWLEDGE OF RESPONDENTS**

22.0 Indicate 1 for ‘TRUE’ or 0 for ‘FALSE’ to the following questions.

| Question number | Statements | True (1) or False (0) |
| --- | --- | --- |
| 22.1 | It is important to eat variety of foods. |  |
| 22.2 | It is of great benefit to consume iron rich foods like organ meat, flesh meat, green leafy vegetables and iron fortified cereals. |  |
| 22.3 | Eating fruits (vitamin C rich contents) increases iron absorption. |  |
| 22.4 | Drinking tea and coffee reduce iron absorption. |  |
| 22.5 | You have heard of iron deficiency anaemia. |  |
| 22.6 | Menstrual blood loss is normal and causes anaemia. |  |
| 22.7 | The following are signs and symptoms of iron deficiency anaemia: extreme fatigue, weakness, pale skin, dizziness, rapid heartbeat, brittle nails. |  |
| 22.8 | Tiredness, loss of concentration and low academic performance are consequences of anaemia. |  |
| 22.9 | Iron/Folic acid supplementation intakes prevent anaemia. |  |

**SECTION D: PRACTICES ON IRON-FOLIC ACID OF RESPONDENTS**

23.0 Indicate 1 for ‘YES’ or 0 for ‘NO’ to the following questions.

| 23.1 | Are you participating in the Girls' Iron-Folate Tablet Supplementation (GIFTS) Programme? |  |
| --- | --- | --- |
| 23.2 | Do you take the iron/folic acid tablet? |  |
| 23.3 | Is the iron/folic acid tablet taken with potable water? |  |
| 23.4 | Do you take iron/folic acid tablet again after a missed opportunity? |  |
| 23.5 | Do you experience hunger, dizziness and weakness after taking the Iron/Folic acid tablet? |  |
| 23.6 | Do you perceive iron/folic acid tablet as a form of contraceptive? |  |
| 23.7 | Does your family encourage intake of Iron/Folic acid supplementation? |  |
